# A comparative analysis of RCC and composite buildings using the new plastic deformation (PD) method

**DOI:** 10.1038/s41598-024-55892-y

**Published:** 2024-03-07

**Authors:** Zhang Qing Qing, Zhang Li Na

**Affiliations:** Department of Jianghe, Architecture College University, Northeastern University, Shenyang, 110819 China

**Keywords:** Civil engineering, Composites

## Abstract

Low computational efficiency and non-linearity behaviour make the simulation of the overall building structure problematic to attain with a single dynamic or static method. Thus, this paper uses a plastic deformation (PD) method based on concrete plasticity theory (CPT) for comparative analysis of multi-storey reinforcement cement concrete (RCC) and composite buildings under common and rare earthquake loads. For this purpose, a 15-storey tall building was selected for analysis using ABAQUS software. At first, a possible building model was created and then plastic deformation analysis was performed using the new PD method under both common and rare earthquakes. After that, a nonlinear time history analysis was conducted, and the results of plastic strain distribution, lateral displacement, peak acceleration, storey stiffness, shear force, storey drift, normalised shear, and top deflection of the RCC and composite buildings were studied deeply. The fundamental time period of the RCC model was found to be 5.2 s while the fundamental time period of the composite model was 6 s. Under common and rare earthquake leads, the peak acceleration of the RCC building was 19% and 22% higher than composite buildings, respectively. Under common and rare seismic loads, the top deflections of the composite building were 33% and 36% higher than those of RCC buildings, respectively. In the case of the RCC building, it was found in this study that higher peak acceleration (PA) of the ground motion led to higher storey top displacement, storey drift, shear force and top deflection under both ground motions. Numerical results suggested that the use of composite structure is more durable than RCC structure. It was also concluded that the PD method could also be effectively used for the analysis of RCC and composite buildings under dynamic loads.

## Introduction

Composite and RCC structures have been fulfilling the demand for buildings all over the world for many years. The construction of the RCC and composite members in the civil engineering field on a large scale shows its importance and confirms its adaptability very well^1,2^. According to structural engineering, composite members can be made by binding effectively two or more than two different types of members together so that they resist loads as a single element^3,4^. Recently, the growing rate of urbanization has seen an increasing trend in the construction of multi-storey buildings worldwide. An essential financial driver for the development of multi-storey buildings is the shortage of land in the heavily urbanised parts of the world^5,6^. Also, the world has acted as another driver for the construction of multi-storey buildings due to the competition for building multi-storey RCC or composite buildings in particular regions^7,8^. Recently, the competition for building the highest has been prolonged to take the challenge of making the spectacular high-rise and most iconic buildings frequently categorized by complex geometries^1,9^.

According to the standard of dynamic design of tall buildings, high-rise buildings can be designed with three-level of fortification targets with two basic design steps. In the first design stage, the elasticity analysis of the building structure under the action of small earthquakes can be carried out based on structural section bearing capacity design^1,10^. To achieve the fortification goal under moderate earthquakes, the structure is required to be rarely encountered with elastoplastic deformation. Because, the irregular and weak parts may cause serious damage to the building structure during the specific earthquake actions. However, the effect of rare (very large) earthquake actions has been judged by performing structural robustness analysis and non-linear dynamic analysis^7,8^.

Previously, many scholars studied the behaviour of tall buildings under seismic loading conditions using different techniques. Such as Papavasileiou et al.^11^ presented a discrete evolution algorithm for optimal structural designs and cost-effective solutions for buildings. Shooli et al.^12^ used a GA-PSO technique for the design and performance analysis of special moment-resisting frames and 2D reinforced concrete buildings. Gholizadeh et al.^13^ presented frame building. Cicconi et al.^14^ proposed a multi-objective optimization technique for the analysis of prefabricated cylindrical steel towers. Di Trapani et al.^15^ utilized retrofitting technique to design RCC columns and beams against dynamic and gravity loads. Also, other scholars have been developed machine learning techniques for tall building analysis under static and dynamic loads^16–19^.

At present, there are two very famous techniques exist to simulate the elastoplastic deformation of buildings under the action of large earthquakes: the seismic elasto-plastic method and the static elasto-plastic method^20,21^. Based on the US seismic evaluation standard, there are two main core theories of the above two methods, one "target displacement theory" and another is "bearing capacity spectrum theory". The bearing capacity spectrum theory has been used to check the elastoplastic failure of RCC or composite structure under the action of rare earthquakes^22,23^. Compared with the current load-bearing capacity theory, the target displacement method can estimate the nonlinear deformation of the structure and its components. Compared with dynamic elastoplastic analysis, input parameters and results of this method (target displacement method) are relatively clear^22,24^. Also, reasonable reinforcement can be estimated intuitively, and relatively less time and cost are required to conduct a more stable analysis adopting the target displacement method. Furthermore, for frame structures, high-rise buildings and complex high-rise buildings, elastoplastic static or seismic analysis methods have been widely used to check the elastoplastic failure of the weak zones of the buildings^25,26^. However, ETABS computer code can be also effectively used to study the seismic and wind effects on multi-storey RCC and composite buildings^27^. SAP 2000 software can analyze and design multi-storey buildings formed by composite members^28^. However, the elasto-plastic method of complex super high-rise and multi-storey buildings to perform calculations under common and rare earthquakes within the framework of ABAQUS software to achieve the fortification goal is still a problem to be solved.

However, this method also has many shortcomings: (1) There is no particularly rigorous theoretical basis. It assumes that the response of a building structure is always related to the equivalent single degree of freedom, and the displacement with the height of the structure is represented by the shape vector. (2) The calculation model of the column and beam using the centralized plastic hinge section can be more accurately simulated, but there has been no ideal calculation model for the shear wall. (3) The horizontal loading mode is not consistent with the actual earthquake action, and the accuracy of the structural target displacement is always compromised. In short, the dynamic effect of an earthquake is approximately equivalent to static load, which can only give the performance of the structure under a certain load but cannot show the actual performance of the building under the specific dynamic load^29^. Also, it cannot correctly consider the lag of the structure under the earthquake. The possibility of structural collapse may not be found precisely. Additionally, the nonlinear dynamic response characteristics such as stiffness degradation and internal force redistribution in the structure due to instantaneous change in an earthquake cannot be calculated with this method^30,31^. Therefore, the static elastoplastic analysis method is only suitable for structures with a small number of layers showing a natural vibration period of fewer than 2 s at the first mode of vibration^21,32^.

In general, plastic analysis of buildings built of elastoplastic materials has increased to fame in structural concept, and the results and methods of their solution have been extensively applied in reality^24^. Elastoplastic study characteristically demands the consideration of inelastic failure that may arise from single or multi-parameter loadings^20,23^. Composite and RCC buildings are important structures mostly constructed in civil engineering, and the plastic response of these high-rise buildings is essential for their overall durability and performance^10^. The importance of this research is to present a novel plastic deformation method that studies the plastic response of RCC and composite buildings by using the strain energy of residual forces in the reinforcing rebars. The main objective of this study is to compare the elastoplastic response of a full-scale RCC and composite buildings by controlling the plastic deformation of the structure. Aiming to find the plastic strain, peak acceleration, displacement, shear force, drift ratio, normalized base shear and top deflection of the RCC and composite tall buildings under common and rare earthquakes.

This article presents a new PD method to compare the different parameters of the multi-storey RCC and composite buildings using finite element software ABAQUS. Under common and rare earthquakes, seismic elastoplastic time history analysis of a 15-storey RCC and composite buildings were performed in this study. Results will provide useful insights to select building types for earthquake proven areas.

### RCC and composite construction

In the past, for the design of a building, the choice was normally between a masonry structure and a concrete structure. However, the failure of many low-rise and multi-storied masonry and RCC buildings due to earthquakes has forced the structural engineers to look for the alternative method of construction^33,34^. Nowadays, the use of hybrid or composite material is of specific interest, because of its important potential to improve the overall performance through rather modest changes in manufacturing and construction technologies. In the world, many consulting engineers are reluctant to accept the use of composite steel–concrete structures because of the unfamiliarity and complexity of its design and analysis. However literature says that if properly configured, then composite steel–concrete systems can provide extremely economical structural systems with superior seismic performance behaviour, rapid erection and high durability^8,35–37^. A need to study the RCC and composite building of the multi-storey keeping given the rapid development in this field is essential. Furthermore, it is comparatively new and no updated analysis methods are available for the same.

## Methodology

In recent decades, researchers from various countries have been developing computer software for structural elastoplastic analysis. DRAIN-2D is the earliest elastoplastic analysis program for plane structures. These computer codes can perform static and dynamic elasto-lastic analysis of multi-storey buildings. The CANNY program uses equivalent beam elements at both ends or the plastic hinges to simulate the behaviour of frames, beams, columns, multi-spring models, shear walls and rigid slabs^38,39^. ETABS and SAP2000 software are also useful for elastic–plastic static analysis, pseudo-dynamic analysis, and dynamic time history response analysis of the structures^40–44^. This software cannot simulate the centralized plastic hinge element of the beam and column more accurately and there is a lack of an ideal calculation model for the elastoplastic analysis of the RCC and composite structure. Also, the horizontal loading mode is not consistent with the actual earthquake action, and the accuracy of the structural target displacement is always compromised in the above software. To overcome the above computational problems, a a new plastic deformation method is developed within the framework of ABAQUS software using Python to study the behaviour of RCC and composite buildings under seismic loads. ABAQUS has good performance in simulating the failure of concrete under cyclic loading and is suitable for dynamic elasticity analysis of complex concrete structures^45,46^. For the concrete damage plastic model, the elastic stage is realized by defining the elastic modulus and ultimate elastic tensile and compressive stress of the concrete. The elastic–plastic stage is determined by the specification for the design of concrete structures^47^.

In this research, the key objective of the PD method is to realize the plastic response of the RCC and composite buildings as it is the stage that should be avoided in the real age of the structure. The complementary strain energy presented below was applied to understand the plastic behaviour of the buildings. This model is applied to steel components that are yielded, and since steel is represented in the composition of this building in two forms which are reinforcing steel bars and steel beam, the steel beam was excluded because it did not yield and the theory was considered to the steel bars used as reinforcement in the column section of the building as they were the only steel elements that yielded.

### Plastic deformation (PD) method

This theory takes into consideration applying a limitation on the plastic deformation created within steel bars by defining the complementary strain energy which was applied and developed by different previous studies^48,49^. The PD method extended the capabilities of the ABAQUS computer code to simulate the behaviour of the RCC and composite buildings. The PD method adopted the plastic deformation theory and tension–compression curve theory for the comparative analysis of RCC and composite buildings under static and dynamic loads. Also, the dynamic analysis method is used for analysing the dynamic response of structures under common and rare earthquakes. Within the framework ABAQUS software, the seismic elastoplastic analysis was first modified by using Python. Then, a multi-storey RCC and composite building were analyzed to judge the performance of the modified method. The plastic deformation method consists on five assumptions: (1) apply seismic wave on the structure as an elastoplastic vibration system; (2) obtain the internal forces and deformation of the structure during ground acceleration; (3) keep the thickness and width of the member so small to control bucking; (4) assume the rigid and plastic behaviour of structural steel; applied different loads for different sections; (5) apply a plastic hinge for a certain dynamic load by applying uniform couple moment.

Consider a structure constructed of an elastic–plastic material that is not affected by time or static-dynamic loads and have a response surface of *S* and a volume of *V*_0_. While a clear portion of *S*_*q*_ and *S* are subjected to quasi-static surface pulls. The other portion *S*_*u*_ is subject to zero structural displacement. The following parameters were explained at response time (*t*), *u*_*i*_(*t*) and ϵ_*ij*_(*t*) are displacements and strain of both structures, respectively. $\sigma_{ij}^{el} (t)$ is pretended stress, which can occur if the building materials are purely elastic. *q*_*i*_(*t*): *σ*_*ij*_(*t*) is shear stresses, $u_{ij}^{el} \left( t \right)$ and $\check{\epsilon }_{{{\text{ij}}}} \left( {\text{t}} \right)$ are pretended displacements and elastic strains, respectively, corresponding to the $\sigma_{ij}^{el} (t)$ parameter. $\tilde{\sigma }_{ij}^{R}$ is a time-independent stress distribution and $\sigma_{ij}^{R} (t)$ is a definite residual stress distribution.

As shown in Eq. (1), the total strain is split into plastic and elastic components. The constitutive principle links the actual stresses to elastic strain portions as,

1 $$ \check{\epsilon }_{{{\text{ij}}}} =\check{\epsilon }_{{{\text{ij}}}} +\acute{\epsilon }_{{{\text{ij}}}} $$

2 $$ \check{\epsilon }_{ij} = \rlap{--} T_{ij} \overline{\sigma } $$

The elastic tensor *Ŧ*_*ij*_ is determined by the related flow rule, and the plastic strain $\acute{\epsilon }_{{{\text{ij}}}}$ is specified by the accompanying flow rule,

3 $$ \acute{\epsilon}_{ij} = \lambda \frac{\partial f}{{\partial \sigma_{ij} }},\lambda \ge 0\;{\text{if}}\;f{\text{ = 0 and }}f = 0{\text{, otherwise }}\lambda = 0 $$

In Eq. (3), *f*(*σ*_*ij*_) is the yield constant and in the stress area, *f*(*σ*_*ij*_) = 0 states a convex surface. The actual stress $\overline{\overline{\sigma }} (t)$(*t*), actual residual stresses $\hat{\sigma }_{ij}$ and fictional elastic stress $\tilde{\sigma }_{ij} (t)$, must satisfy the following Eq. (4):

4 $$ \overline{\overline{\sigma }} \left( t \right) = \tilde{\sigma }_{ij} \left( t \right) + \hat{\sigma }_{ij} $$

In Eq. (5), $\hat{\varepsilon }_{{{\text{ij}}}} \left( {\text{t}} \right)$ is the fictitious elastic strain and by the constitutive law it can be determined as

5 $$ \check{\epsilon }_{ij} \left( t \right) = \rlap{--} T_{ij} \overline{\sigma }(t) $$

Calculate the overall corresponding plastic work *W*_*p*_(*τ*) produced along a load route from *t* = 0 to *t* = *τ*. This study may be used to evaluate an elastoplastic structure’s overall plastic deformation and plastic performance. Also, the next theorem is selected here to find its upper bound and self-stress distribution $\tilde{\sigma }_{ij}^{R}$ can meet the condition:

6 $$ f\left( {\sigma_{ij}^{E} \left( t \right) + \tilde{\sigma }_{ij}^{R} } \right) \le 0, $$

This meets in *V* at any time *t* ≤ τ, then the subsequent condition arrays the upper constraint on the entire corresponding plastic work:

7 $$ W_{p} (\tau ) \le 0.5\smallint \rlap{--} T_{ij} \tilde{\sigma }_{ij}^{R} \tilde{\sigma }_{kl}^{R} dV $$

It should be emphasized that the limit can improved by picking the factual value for $\tilde{\sigma }_{ij}^{R}$. Enact a constraint (*W*_*p*0_) on the plastic work (*W*_*p*_) to prevent extreme plastic deformations of both structures. The limits of the plastic deformations were define through residual stresses; in contrast, it is supposed that:

8 $$ \tilde{\sigma }_{ij}^{R} \equiv \sigma_{ij}^{R} $$

This presumption gives a reasonable upper bound and helps us to formulate the problem properly. In such case, the plastic deformation constraint will be like the following:

9 $$ W_{p} (\tau ) = 0.5\mathop \int \limits_{{V_{0} }}^{V} \rlap{--} T_{ij} \sigma_{ij}^{R} \sigma_{kl}^{R} dV - W_{p0} \le 0 $$

Consequently, a new computational model is developed by using limitations for energy quantities to define the corresponding strain energy of residual forces as a plastic behaviour of both RCC and composite structures. For the example of structural elements including steel bars, Eq. (9) was developed, and the residual forces were used first time to simulate the strain energy as:

10 $$ W_{p} = \frac{1}{2E}\mathop \sum \limits_{i = 1}^{n} \frac{{l_{i} }}{{A_{i} }}N_{i}^{{R^{2} }} \le W_{p0} $$

where *W*_*p*0_ is allowable energy for *W*_*p*_, and *l*_*i*_, (*i* = 1, 2, … , *n*) represents the length of the element., *A*_*i*_,(*i* = 1, 2, …, *n*) is the cross-sectional area of the members and ${N}_{\text{i}}^{\text{R}}$ is the residual force of the elements. *E* is the modulus of elasticity of the materials.

In addition, when the load *P*^0^ is applied, the internal elastic force *N*^*el*^ and the internal plastic force *N*^*pl*^ show the residual forces *N*^*R*^ that remain in both structures after the unloading is complete:

11 $$ N^{R} = N^{pl} - N^{el} $$

where

12 $$ N^{el} = F^{ - 1} G^{T} K^{ - 1} P_{0} $$

In Eq. (12), *F*, *G* and *K* are flexibility matrix, geometry matrix and stiffness matrix, respectively. The limit of plastic deformation is considered to be steel bars placed inside the reinforced concrete columns and beams of the RCC structure. Internal forces developed in concrete are not included in the simulation process due to its low effect on tension in comparison to steel, while it is recognised that steel endures more tension strain than concrete, which can cause an early collapse of concrete subjected to tension.

**Figure 1:**
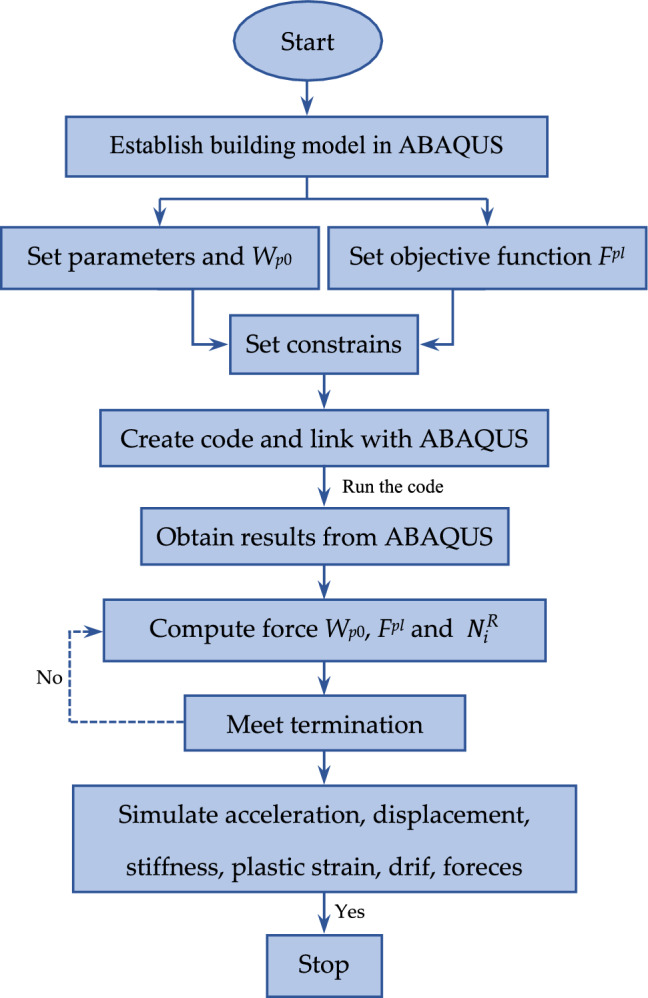
Calculation procedure of the PD method.

Moreover, in order to promote the extensive application of the new PD method, this paper combined the idea of PD with the finite element software ABAQUS. The advantages of ABAQUS software in terms of non-linear calculation and visualisation were fully utilised to realise ABAQUS-based PD analysis. Also, the poor convergence of the non-dynamic simulation diminishes the computational efficiency of the deformation analysis method. This is because of the main analysis that undergoes excessive plastic yielding which leads to convergence problems and solution inaccuracy. Also, unstable material behaviour, insufficient material data with respect to stress–strain data, insufficient mesh refinement and unstable deformation, such as buckling^50,51^. In order to improve the convergence of the non-dynamic simulation of such RCC and composite structures, this paper suggested a new static-dynamic analysis (PD) method with the aim of improving the convergence and reducing the degrees of freedom. The above calculation process was combined with the finite element software ABAQUS as shown in Fig. 1.

### ABAQUS computation

The ABAQUS Scripting Interface (ASI) is a Python-based interface that allows users to automate ABAQUS simulations by a bew writing scripts. In this research, ASI was used to integrate the above equation system into the ABAQUS model. Specifically, a programming script that defined the objective function, constraints and the nonlinear problem was considered in this comparative study. All forces and moments were calculated after running the code for all the increments in order to determine the optimum load (Fig. 1). The corresponding plastic strain was simulated and matched to the allowable value stated in the software. ABAQUS computer code was used in this study to create the finite element model under static and dynamic loads, and tension–compression curve theory was adopted for concrete material^52^. After that, a fixed region technique was selected to insert the steel stirrups and bars into the concrete. The friction between the segmental joint and the bottom of the column (RCC or composite) is selected by surface to surface contact model. Tensile damage variables and compression damage variables of concrete were adopted from the Minh et al.^53^ study.

The effective stiffness of a member is the slope of a line between the origin and any point on the skeleton curve. Also, this is the ratio of a force (*F*_*i*_) in horizontal direction and coordinates of a point on a horizontal surface, which can be expressed in Eq. (13)

13 $$ K_{s} = \frac{{F_{i} }}{{\Delta_{i} }} $$

where: *F*_*i*_ is the negative and positive ultimate load of the storey at *i*th level of load level, and *∆*_*i*_ is the negative and positive ultimate deformation of the storey at *i*-th level of load.

## Finite element modeling

Many scholars used the finite element method to analyse behaviour of tall buildings^25,54^. So, the use of appropriate modelling methods is essential for the precise investigation of tall buildings under the influence of earthquake loads. In this study, a 2D full-scale finite element model has been used to check the performance of RCC and composite buildings. Both models were designed using the multi-storey building analysis program ABAQUS. To make sure that both structures (RCC and composite) in current research are precisely similar to the conservative concepts. These tall buildings are designed to withstand live, dead and seismic loads.

### Model parameters

**Figure 2:**
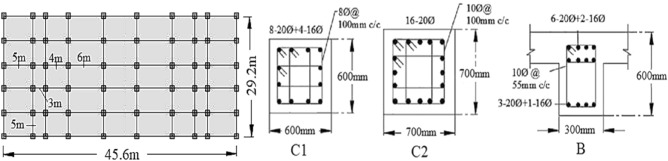
Planar diagram of the structure and reinforcement detail of columns and beam.

**Figure 3:**
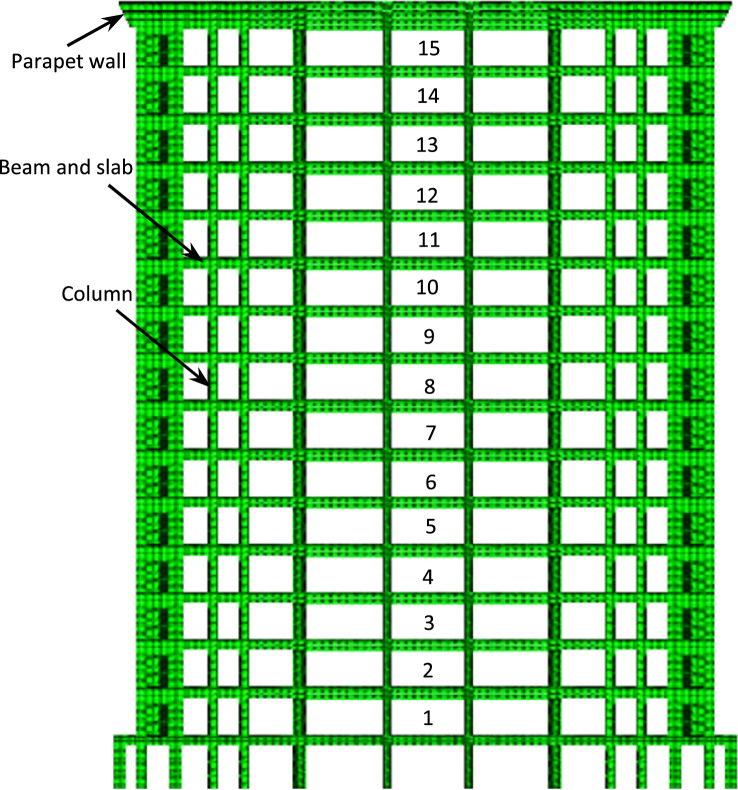
Finite element model.

Composite construction helps to define structural elements covered in concrete-filled steel cassions in the case of beams, column and roof slabs. These are made of different steel sections with shear connectors so that these members will resist load together as a single member. In this study, a 15-storey RCC and composite building is selected for comparative analysis. Figure 2 shows a building plan with 5 × 6 bays and a distance between columns. The size of columns, beam and detail of reinforcement is given in Fig. 2. Considering the standards of dynamic design of structures^29^, the seismic fortification intensity of the super high-rise structure was 9 degrees. The design's basic seismic acceleration was 0.45 g. The length and width of the building are 45.6 m × 29.2 m, respectively (Fig. 2). There are total of 15-storeys of the building and the height of each storey is 3.3 m (Fig. 3). Each floor plan, loading conditions and other building details are given in Table 1. Loading conditions for both RCC and composite buildings are supposed to be the same. The total height of the structure is 49.5 m, which exceeds the maximum applicable height of A-level high-rise structures.

**Table 1:** Input parameters of RCC and composite structure.

Description	RCC structure	Composite structure
Plan dimension	45.6 × 29.2 m	45.6 × 29.2 m
Height of building	49.5 m	49.5 m
Height of each storey	3.3 m	3.3 m
Height of parapet	1.5 m	1.5 m
Depth of foundation	5 m	5 m
Size of column C_1_	700 × 700 mm	CFST
Size of column C_2_	600 × 600 mm	CFST
Size of beam	300 × 600 mm	ISMB 450
Thickness of slab	200 mm	200 mm
Seismic zone	III	III
Importance factor	1.0	1.0
Wind speed	60 m/s	60 m/s
Zone factor	0.15	0.15
Damping ratio	5%	5%
Seismic intensity	9^o^	9^o^
Floor finish	1.0 kN/m^2^	1.0 kN/m^2^
Live load	5.0 kN/m^2^	5.0 kN/m^2^
Density of concrete	25 kN/m^3^	25 kN/m^3^
Density of brick	20 kN/m^3^	20 kN/m^3^
Density of steel	7850 kN/m^3^	7850 kN/m^3^
Grade of concrete	M20	M20
Grade of steel	Fe415	Fe415

A 15-storey building with common geometry and dimensions that are used usually in construction is selected to conduct comparative analysis (Fig. 3). all structural elements were modelled using ABAQUS software to analyse the behaviour of multi-storey RCC and composite buildings as shown in Fig. 3. The height of each floor is 3.3 m. Two types of lateral load resistance systems, including moment frame and moment frame with inversed V centrically braced system, were considered for composite building. For all models, the concrete slabs with a thickness of 200 mm are selected. Furthermore, 2UNP, I-shape beam, and BOX profiles were used for braces, beams, and columns, respectively. The dimensions and parameters of the building model are provided in Table 1. The beams, columns and steel bars were separately modelled using solid C3D8R elements, T3D2R elements and REBAR elements. Then these components were coupled with single degrees of freedom using EMBED technology.

In most studies and experiments it has been seen that with the sudden failure of building key elements (beam and columns), the structure behaviour enters the plastic region. The model possesses non-linear analysis, non-linear geometric behaviour and non-linear material property. With a Von Mises yielding principle, the isotropic hardening law was selected here to simulate the plastic deformations of the building components. The true stress–strain graph is entered into the ABAQUS to describe the steel plastic parameters. The unit weight of each member was defined with the mass density option. To define the elastic phase of steel, Young's modulus and Poisson's ratio of 2.2 × 10^5^ N/mm^2^ and 0.33 were selected, respectively. The yield stress of 370 N/mm^2^ is applied for all steel material. The plastic option was used to define the plastic portion of the stress–strain curve. Steel grade ST37 was applied for all the structural steel. The concrete’s constitutive behaviour is modeled by a three-dimensional continuum, the plasticity damage model.

The concrete damaged plasticity model may model concrete in all element types including columns, beams and slabs. Inelastic actions of concrete are presented via isotropic damaged elasticity theory along with the isotropic tensile and compressive plasticity. The concrete unit weight value was 2500 kg/m^3^ and the compressive strength of concrete was 29 N/mm^2^. Conventionally, the tensile cracking stress was roughly supposed 5.7% of the peak compressive stress.

### Meshing element type

Since the models of this study have many components and are full-scale modeled, so the beams, columns, and braces are simulated employing the C3D8R element. Using this element, a large amount of structural calculation can be reduced well^54^. The slab is modelled using the S4R element, which has six degrees of freedom per node and four nodes. From the ABAQUS library, the REBAR element was used to define reinforcement in each member by outlining the reinforcement area at the appropriate depth of the cross-section. The main reinforcement included is the A252 mesh assumed to act 20 mm from the top of the slab and 20 mm thick at the bottom. This reinforcement is defined in both directions of the slab. Mesh sensitivity analysis was also executed to define the size of the mesh in each section of the building. For this purpose, an appropriate mesh size of 30 mm for beam, column and slab was selected in numerical coned. The contact between the beam, column and slab is defined by “tie constraint”. In this contact technique, the nodes of the beam cross-section are constrained to the nodes of slab edges. Furthermore, the connections of the elements of the composite building were considered rigid and join connectors defined by braces to columns and beams connections.

### Generation of earthquake

Earthquake normally consists of typical randomness in intensity, space and time, and their excitation mechanism is regarded as a stochastic process^55,56^. Thus, the standard of the stochastic process can be selected to construct the desired earthquake excitation. The spectral representation technique is one of the most commonly accepted approaches^57^ when the harmonic waves were superposed to simulate the random process. The previous studies of stationary processes ignores the effect of site conditions. With the development of earthquake theory, the effect of earthquake non-stationarity in the dynamic input is further excavated by scholars, and the time-changing characteristics in frequency and intensity could greatly affect the structural performance as well as the earthquake property^58,59^. In this study, the spectral representation method is used to generate the common and rare earthquakes according to the method presented by Xu et al.^55^.

### Accuracy and validation

To check the accuracy of the proposed approach, the determination coefficient (*R*^2^) has been largely used and also is well known today. The *R*^2^ value explains the goodness of any method, which is a way to observe the accuracy of a method in anticipating the factual data sets. A higher value of *R*^2^ shows that the calculating precision of the method is high. These matrices used the following Eq. (14) as:

14 $$ {\text{R}}^{2}  = \sum\limits_{{{\text{i}} = 1}}^{{\text{n}}} ( \tau _{{\text{i}}}  - {{\bar{\text{O}}}})^{2} /\sum\limits_{{{\text{i}} - 1}}^{{\text{n}}} ( \tau _{{\text{i}}}  - {{\bar{\text{O}}}})^{2}  $$

where *τ* and *Ō* are the targeted and output values, respectively, and *n* is the number of specimens.

**Figure 4:**
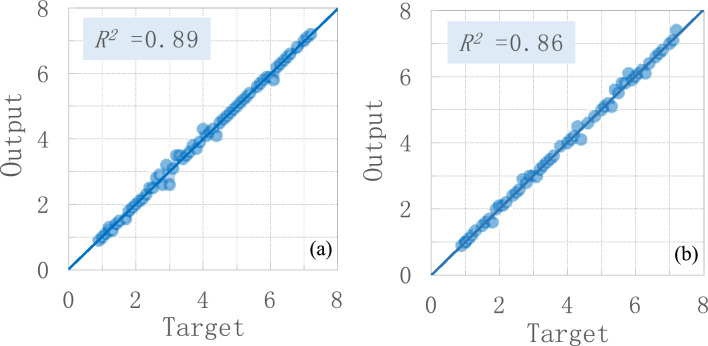
Accuracy of the proposed model: (**a**) RCC building (**b**) composite.

A comparison of targeted and output values is presented in Fig. 4, at the calculated and simulated stage. The constant of determination (*R*^2^) between the calculated and simulated results shows a good analysis capacity of the proposed model. There is almost no remarkable dissimilarity between the calculated and simulated results (Fig. 4). Results show that the developed ABAQUS-based numerical model is an appropriate tool to analyse RCC and composite buildings under common and rare earthquake loads.

A large experimental model is too difficult to assume the structure’s displacement and distribution of plastic strain under common and rare earthquakes. ABAQUS software is a great option for examining the problems such as the distribution of plastic strain. Using the finite element method within the framework of ABAQUS, it is possible to consider different sorts of models if the models assumptions, elements step analyses and contacting prescriptions are properly validated by numerical simulation.

## Results and discussions

After creating a building model in ABAQUS software, plastic strain, peak acceleration, inter-storey displacement under common rare earthquake, displacement and shear force history curves of elastic and elastoplastic analysis under rare earthquakes, plastic deformation distribution under rare earthquakes and plastic strain of coupling beam of an RCC and composite building were compared in this section.

### Plastic strain

**Figure 5:**
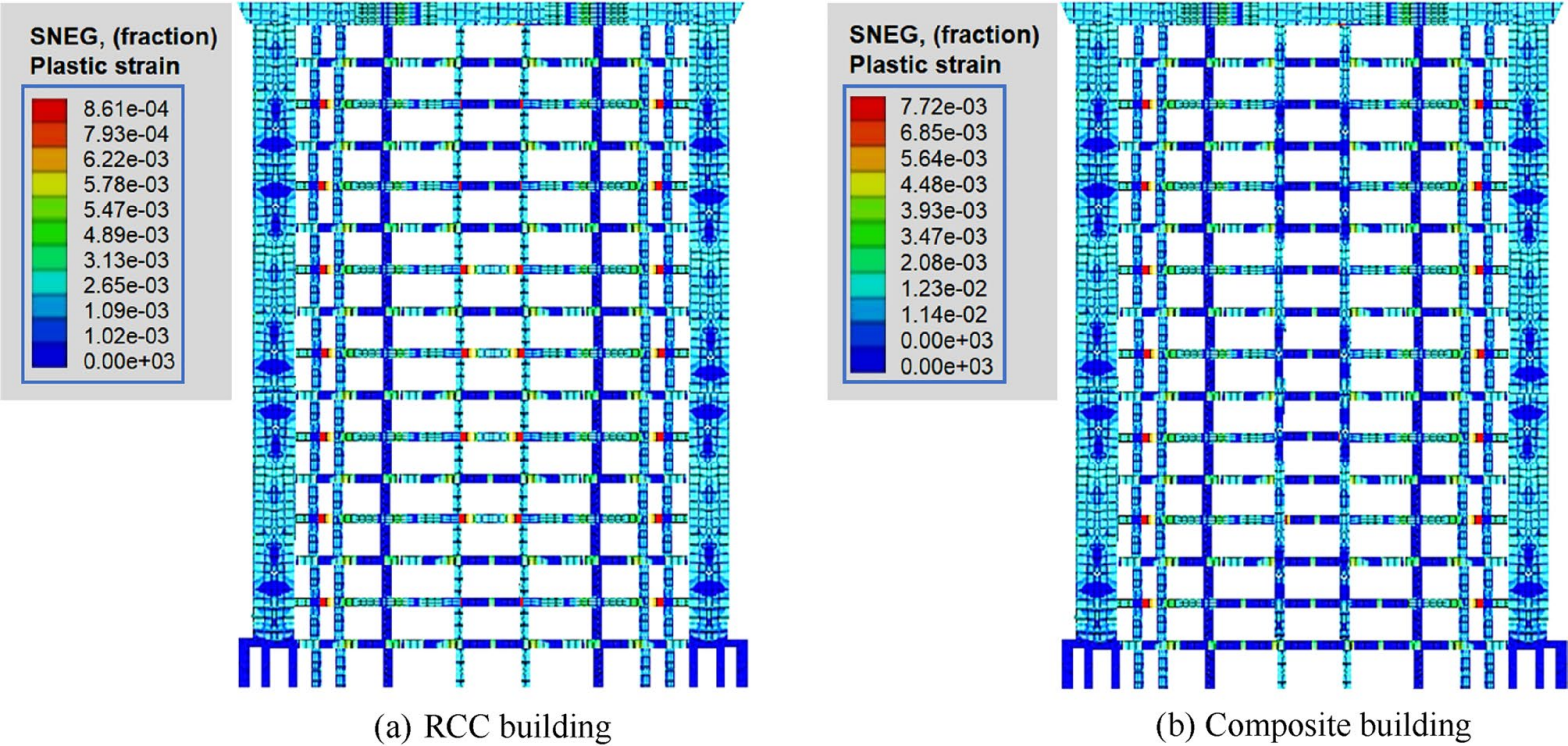
Distribution of plastic strain under common earthquake.

**Figure 6:**
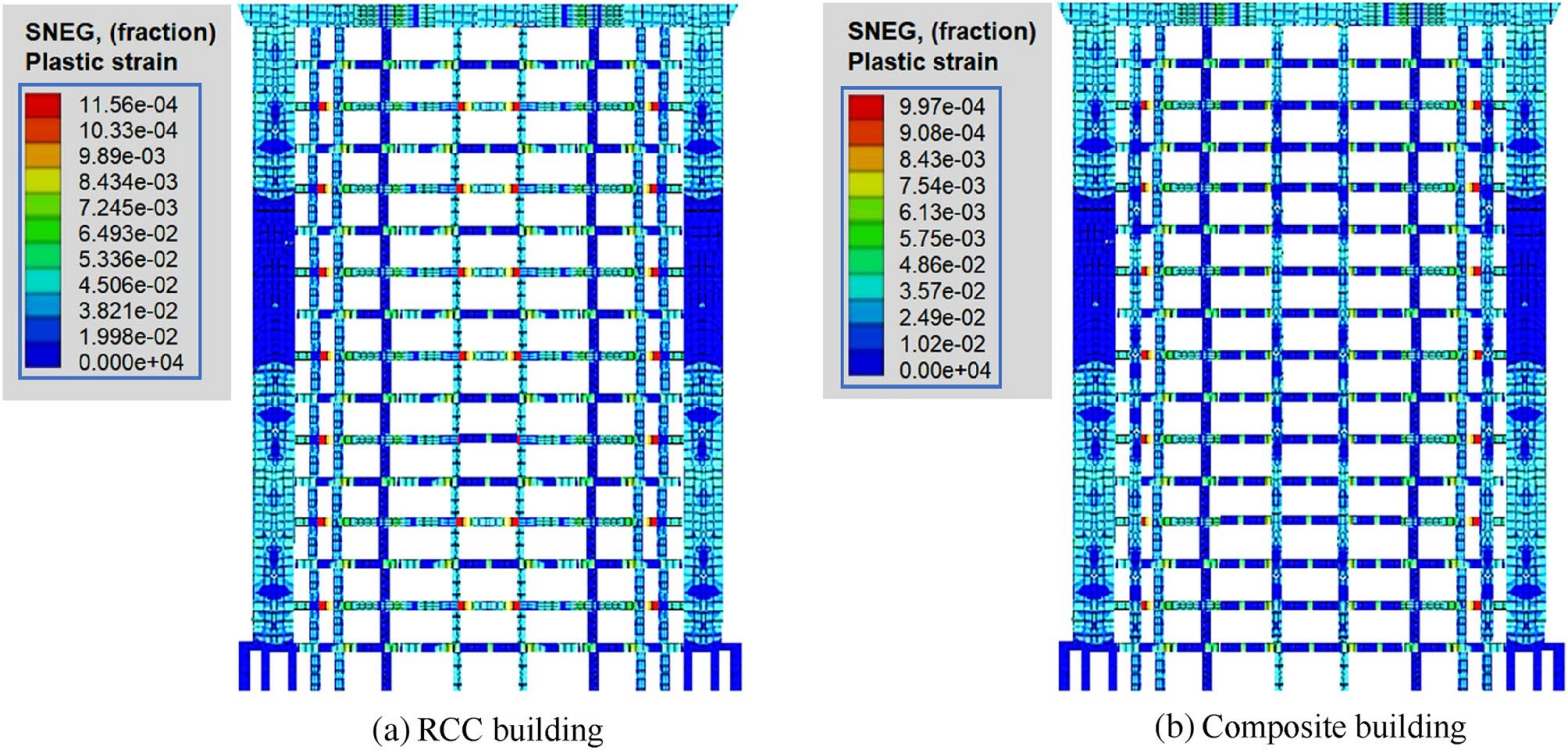
Plastic deformation under rare earthquake.

A plastic strain contour map is a parameter which displays the element strain rate when translated from a point to another points or falling. Figure 5 shows the distribution of plastic strain under the effect of common earthquakes for RCC and composite models. By comparing models of RCC with the composite model observed that a maximum plastic strain of 7.93 × 10^–4^ was produced in the case of a common earthquake (Fig. 5a). However, the maximum plastic strain of 6.85 × 10^–3^ for the composite model under common earthquake produced in the beams of the structure as shown in Fig. 5b. In the case of RCC and composite buildings, when a common earthquake load was applied, the joints of beams and columns vibrate and show the maximum magnitude of plastic strain of 8.61 × 10^–4^ and 7.72 × 10^–3^ 9.7 produce, respectively (Fig. 5). As indicated in Fig. 5a, b, the maximum plastic strain also in central columns of RCC building, but this phenomena does not observed in case of composite building. Under rare earthquakes, the maximum plastic strain of 10.33 × 10^–4^ and 9.1 × 10^–4^ was produced in both RCC and composite buildings (Fig. 6).

Also, the behaviour of coupling beams is presented in Figs. 5 and 6. According to the results of the plastic analysis, the plastic hinge first appeared at the end of the connecting beam of the longitudinal seismic wall under a rare earthquake. The effective plastic strain clearly shows the distribution of the plastic hinge (Figs. 5 and 6). It can also be observed from the analysis of plastic strain that the plastic deformation of most connecting beams starts after 2nd floor. Mostly connecting beams enter the plastic state. Subsequently, plastic regions do not appear in the upper and lower parts of the structures. Plastic hinges mostly appeared at the joints of beams and columns. At both ends of the connecting beams, the equivalent plastic strain reaches the maximum value under both common and rare earthquakes.

From the overall point of view, the value of the plastic strain in the RCC structure was higher as compared to compiste building. As compared to the RCC builiding, fewer numbers of connecting beams enter the plastic state plastic in the case of the composite structure. Furthermore, under the action of rare earthquake, considering the second-order effect of gravity and large deformation, the composite structure has no large area of plastic damage except a few coupling beams (Fig. 5). But, the plastic deformation of some coupling beams under rare earthquake is too large, such as the maximum equivalent plastic strain of coupling beams after 1st floor was 9.97 × 10^–4^ (Fig. 6).

The motions that resulted in the vertical crushing of the structure, in general, correspond to larger flexural deformation ratios in the bottom stories. This is attributed to two reasons: first, the shear reinforcement ratio in the x-direction is larger than that in the y-direction; second, given that the plastic deformation ratio is calculated for the columns and beams that resist the large shear force, and the plastic deformation ratio in the x-direction parallel to the column that shows large axial compression due to the coupling action of the beams. Furthermore, the shear deformation ratios in the RCC models are generally larger than those of composite models under both common and rare earthquakes. Results of plastic strain suggest that the proposed method (PD) can perform plastic analysis of RCC and composite buildings subjected to large dynamic loads. It is logical since PD investigation has confirmed that the composite structure may exhibit more nonlinear stiffness because of its limited energy dissipating capacity.

### Peak acceleration

**Figure 7:**
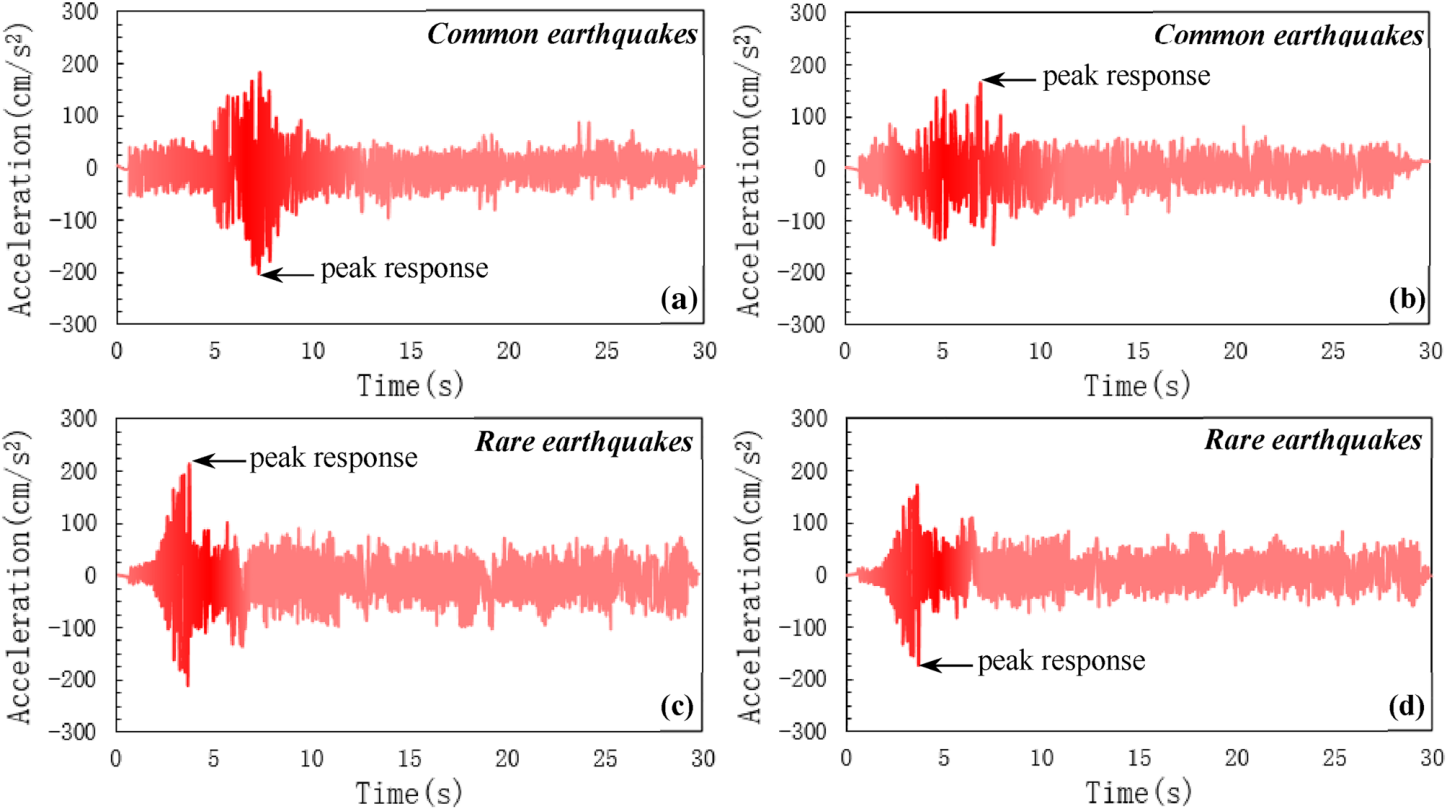
Buildings peak response time under common and rare earthquakes (**a**, **c**) RCC building and (**b**, **d**) composite building.

To simulate the response of both buildings, a time history analysis is performed under an earthquake record from the past. The dynamic hazard level of the buildings was assumed to be zone III (PGA-0.24 g). Far-field and near field ground motion were selected from Saha and Mishra’s^60^ study. The ground motions were applied simultaneously in the *x* and *y* directions. The peak acceleration of the RCC and composite buildings is studied by the relationship between the peak acceleration and time. The accelerograms of the ground motions have been shown in Fig. 7. It is clear from Fig. 7, that the peak ground acceleration is higher in RCC buildings as compared to composite buildings. Under common earthquakes, the peak acceleration of 200 cm/s^2^ in the case of the RCC building was recorded without frequency modulation at a seismic wave duration of 7 s (Fig. 7a). Figure 4(b) shows the peak acceleration of the composite building. The maximum peak acceleration of 170 cm/s^2^ was recorded at a seismic wave duration of 7 s (Fig. 4b). At common earthquake-loading conditions, the peak acceleration of the RCC building was 19% higher than composite building at almost the same peak seismic response time.

Figure 7c, d shows the peak response of both RCC and composite buildings under rare seismic loads. The peak acceleration of the structure composed of reinforcement concrete is 200 cm/s^2^ a response time of 4 s (Fig. 7a). At a structural response time of 4 s, the maximum acceleration of 170 cm/s^2^ was recorded when rare earthquake load is applied to model (Fig. 7d). At rare earthquake loading conditions, the peak acceleration of the RCC building was 22% higer than composite building at almost same peak seismic response time. Under common earthquakes, the response time of both buildings was higher as compared to rare dynamic loads. Furthermore, the acceleration amplitude was reduced by adding composite members but the peak acceleration reached 190 cm/s^2^, which may disturb the comfort and resistance of the building. On the other hand, the RCC mode of construction is less efficient in reducing the acceleration amplitude by observing the time response of the top floor acceleration of a 15-storey building subjected to common and rare earthquake forces.

### Displacement history

**Figure 8:**
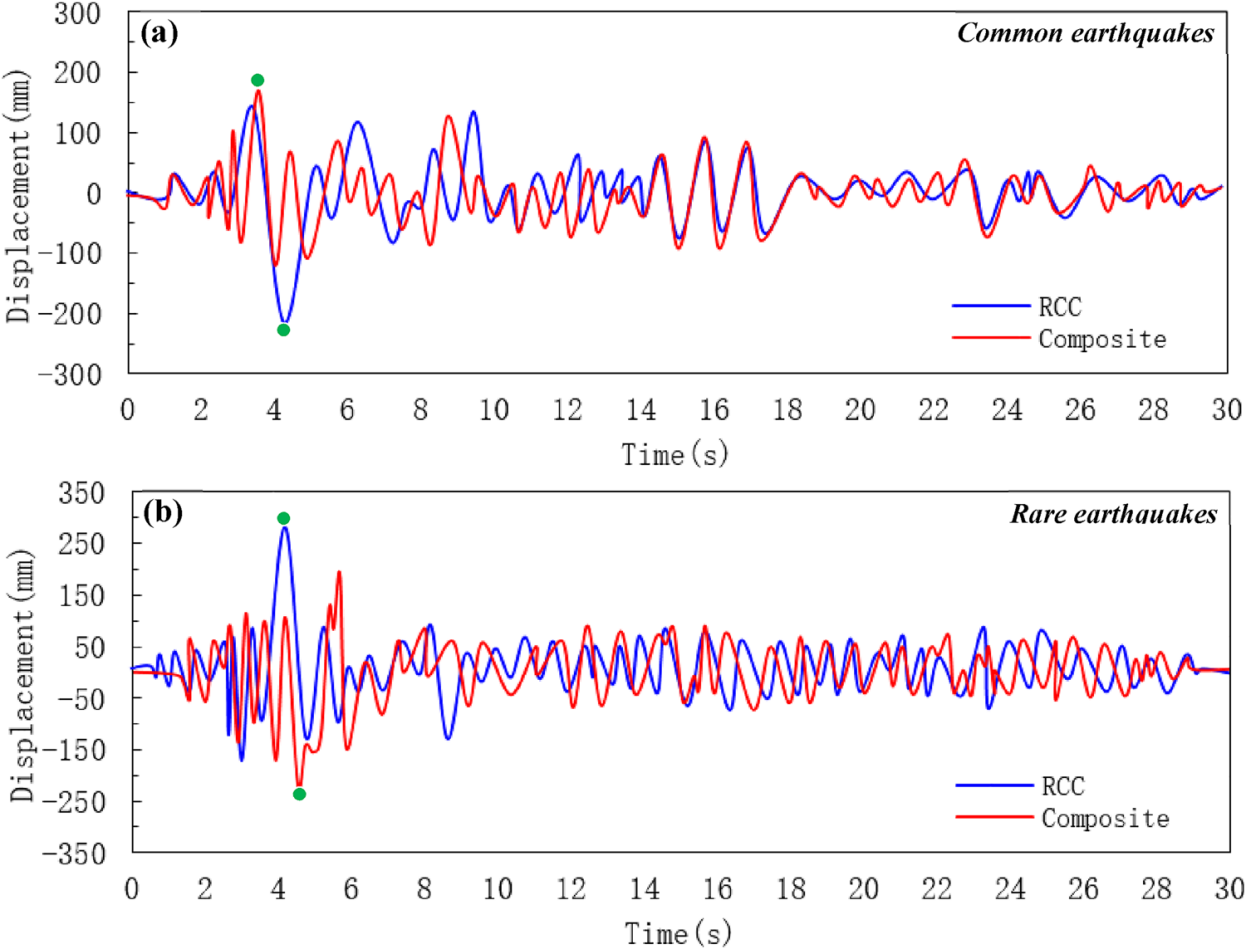
Displacement history curve of RCC and composite building. (**a**) under common earthquakes, and (**b**) under rare earthquakes.

Structural failures are sustained by lateral displacements^61^. A building deforms significantly when it is subjected to strong seismic loads. Under common and rare seismic loads, a dynamic time history analysis was selected to simulate the maximum displacement of each floor of the buildings. Figure 8 shows that displacement in the top floors is greater for each storey than that in the base floors of the building. Under the common earthquake action, the displacement of RCC and composite models increased as the response time rose and the maximum displacement of 240 and 180 mm is observed at 5 s, respectively, as presented in Fig. 8a. In case of a common earthquake, the storey displacement suddenly rose after the 4th floor and almost maintain the same value up to 15 floors. The displacement of RCC and composite structure almost linearly increased under rare earthquakes. Because the rigidity of the structure was less than that of the 4th floor, so the inter-storey displacement suddenly increased on the 4th floor, and the structure changed from the 5th floor or above.

The overall displacement in lower floors was small as compare to the upper floors. The maximum displacement between the top floors was more, but the displacement value between floors under the "large earthquake" did not exceed the "Code for Seismic Design of Buildings". Under rare earthquakes, the maximum displacement of the RCC and composite buildings are 300 and 220 mm at the response time of 4 s and 4.3 s, respectively (Fig. 8b). The comparison of the displacement history displays that the RCC structure shows the elastoplastic deformation at 4 s of the seismic wave input. However, the composite building enters into the elastoplastic deformation at 5.55 s of the seismic wave. Composite buildings showed more resistance under earthquake loading as compared to RCC buildings.

In addition, the responses of the two systems are almost similar under minor vibration, which is treated as linear responses in Fig. 8. The peak displacement of the two models is not the same. This situation is particularly taken to reduce the effect of the peak displacement responses. Generally speaking, compared with composite buildings, RCC buildings may display maximum displacement responses because of its small energy-dissipating ability. But, the peak response of both structures appears at the beginning of the nonlinear vibration process. In this case, the strength and stiffness of the structure control the peak response. Conversely, the response of both buildings is entirely dissimilar after approaching the peak displacement of 300 mm. The nonlinear performance of an RCC mode of construction is mostly decided by the plastic performance of energy-dissipating structural members. The energy-dissipating ability of RCC buildings under nonlinear displacement is comparatively not enough compared with those of composite mode of construction.

The peak transient response points are marked in Fig. 8. Note that the peak transient inter-story displacement of each story does not arise at the same time by PD analysis. However, the peak response points are all included in a great nonlinear response process, as marked in Fig. 8. Figure 8a, b presents the inter-story responses of RCC and composite buildings under common and rare earthquakes. It can be noted that the inter-story responses of both buildings remain important during the whole nonlinear behaviour and the maximum inter-story responses show in this process. It is understandable since PD analysis has validated that the RCC may exhibit more nonlinear stiffness due to its limited energy-dissipating capacity^62^. Prior investigators have also mentioned that the fact that the inelastic state of the system may greatly affect the higher mode effect^63,64^. Generally speaking, compared with a composite building, an RCC building with the same stiffness and strength may exhibit a greater response due to its limited energy-dissipating capacity. Thus, the RCC system may exhibit much more nonlinear behaviour.

### Shear force

**Figure 9:**
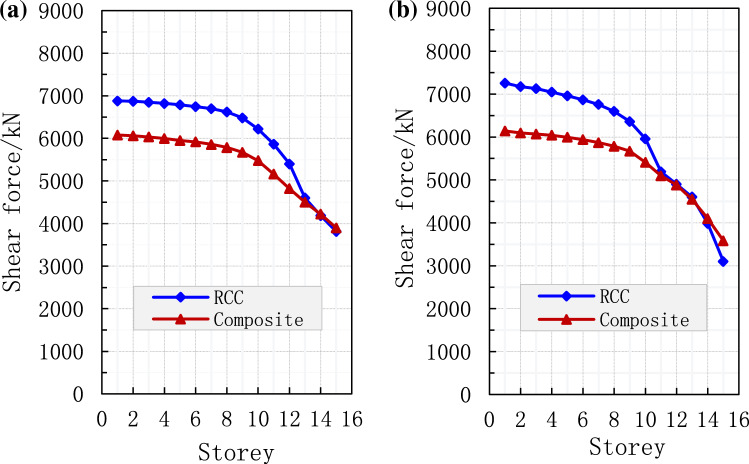
Comparison of shear force in each storey, (**a**) under common earthquake and (**b**) under rare earthquake.

The shear force in each storey of both structures is also studied by ABAQUS software at the same material properties. Under common and rare earthquakes, the relationship between shear force and building storey is presented as shown in Fig. 9. As the number of storeys rose, the shear force decreased significantly in both cases (Fig. 9). Under common earthquake, the maximum shear force in RCC and composite buildings were 6800 and 6200 kN, respectively (Fig. 9a). The shear force in the RCC and composite structures was decreased to 79 and 58%, respectively, when the model was subjected to common earthquake (Fig. 9a).

In the case of rare earthquakes, the maximum shear force in RCC and composite buildings were 7400 and 6300 kN, respectively (Fig. 9b). Concerning floor number, the shear force in the RCC and composite structures was decreased to 100 and 57%, respectively, when the model was subjected to common earthquake as shown in Fig. 9b. Furthermore, the overall shear force in RCC structure was higher under common and rare earthquakes.

It can be noted that RCC structure requires more force demands along the height, compared with composite buildings, especially for top and bottom stories. The similar shear force for the two different models in this study is caused by the same response factor for the first model shear force demands. Compared with the composite model, RCC requires more design force in upper stories for the considered building. The shear force demand of top stories was less as compared to base stories. It can be noted from the results of PD analysis that the initial shear force demands of higher modes for mid-height stories are always lower than those for top floors.

### Drift ratio and displacement

**Figure 10:**
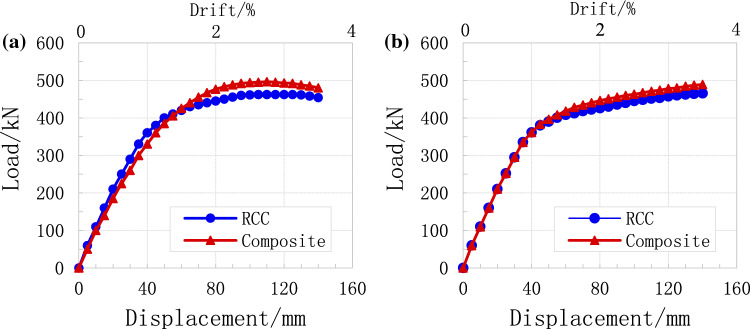
Comparison of overall displacement and drift%, (**a**) common earthquake (**b**) rare earthquake.

The difference between the lateral displacement of two consecutive stories is known as inter-storey drift. This is a very important parameter which influences the stability of the buildings and the comfort of the occupants during and after earthquake motion^65^. Figure 5 shows the inter-storey drift ratio under near-field and far field ground motion. The inter-storey drift ratio was calculated by taking the difference between the storey displacement of two consecutive stories and dividing it by the height of the storey^65^. Under common dynamic loads, the displacement and drift ratio of the RCC building was higher (Fig. 10a). As the dynamic load increased from 0 to 400 (kN), the building displacement and drift increased sharply up to 40 cm and 1.0 (%), respectively, as shown in Fig. 10a. After that, the displacement and drift rose very slowly up to 57 cm and 1.7 (%), respectively. At last, the displacement and drift start to decreased slowly. In the case of composite building, as the load increased the displacement and drift rapidly rose up to 30 mm and 0.8% (Fig. 10b). After that the displacement and drift slowly increased as shown in Fig. 10b. The numerical results and experimental results were almost similar, which shows the accuracy of the proposed method.

According to numerical results, composite members are more durable and resist more loads as compared to to RCC structures under both static and dynamic loading conditions. However, many scholars and designers are not welcoming the construction of composite multi-storey buildings due to their complex design analysis, unfamiliarity, and lack of awareness^66,67^. On the other hand, the desirability of composite mode construction for tall buildings is due to its effective shear connection between the RCC and structural steel. This is the shear connection that permits both static and dynamic forces to transfer easily in other members^68^. So, shear connections are very important and very basic members for composite construction under static and dynamic loads. Shear connections increase the load resistance ability and overall rigidity of the structural elements (*e.g.,* column and beam).

### Storey stiffness

**Figure 11:**
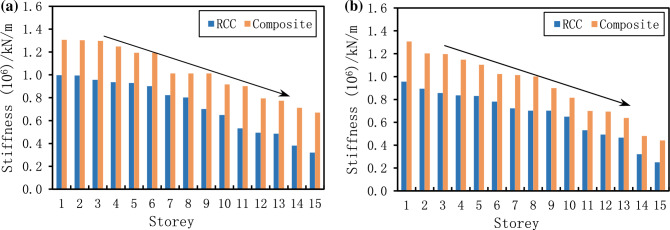
Comparison of storey stiffness under different earthquakes, (**a**) under common earthquakes and (**b**) under rare earthquakes.

During strong earthquake shaking, stiffness irregularity along the height of the building leads to undesirable effects, including localized lateral deformation and the formation of undesirable collapse mechanisms in buildings^69^. The storey stiffness of structures is studied through finite element software under common and rare earthquakes. The stiffness of each storey has been given in Fig. 11. From the finite element analysis, it was observed that the overall stiffness of each storey in the case of the composite structure was higher as compared to RCC structure under common and rare earthquakes (Fig. 11). Under common earthquake, the stiffness of RCC and composite structure decreased 67 and 62%, respectively, as the number of stories increased (Fig. 11a). In case of rare earthquake, the stiffness of RCC and composite structure decreased 73 and 66%, respectively, as the number of stories increased as shown in Fig. 11b.

Under rare earthquakes, the average stiffness of the RCC and composite structure was 0.665 × 10^6^ (kN/m) and 0.911 × 10^6^ (kN/m), respectively. Also, in the case of a rare earthquake, the average stiffness of the RCC and composite structure was 0.727 × 10^6^ (kN/m) and 1.023 × 10^6^ (kN/m), respectively. Results of Fig. 11 suggested that the composite structures are more stiff and more suitable for construction under both common and rare earthquakes. Because composite buildings resist more lateral deflection at lateral force. Also, results showed that the proposed method can simulate the storey stiffness of different buildings under different earthquake scenarios.

### Normalised storey shear

**Figure 12:**
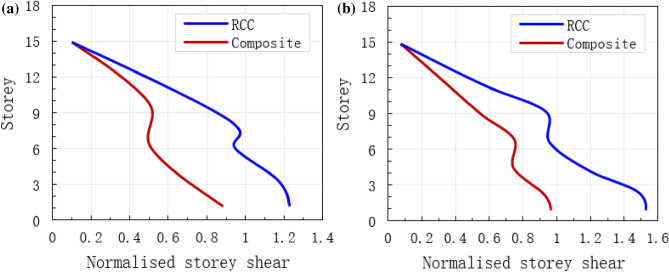
Normalised storey shear of both RCC and composite buildings, (**a**) under common earthquake and (**b**) under rare earthquake.

Normalised storey shear for the two buildings under the considered earthquakes are shown in Fig. 12. Storey shears are normalised with the total weight of the structure, W = Σm_j_ × g. Between both structures, at different numbers of storeys and earthquakes, it is observed that the composite mode of construction shows the least normalised storey shear which evidently confirms the effectiveness of the new method. The peak normalised shear of the two buildings with 15 storeys under different earthquakes is presented in Fig. 12. Under common and rare earthquakes, It is observed from the analysis that the peak normalised base shears were more in RCC building as compared to composite building, except for first two floors where the fundamental modal time period of structure is in acceleration dominant zone of earthquake response spectrum (Fig. 12). The reason is that the RCC building resists less lateral deflection at different forces. Also, RCC exhibits the least seismic response at common and rare earthquake loads with a reduction ranging from 15.8 to 45.3% as compared to the composite model. The results of the normalised base shear for both buildings were appropriate, which shows that the proposed method has the ability to analyse the normalised base shear of the buildings.

### Lateral displacement

**Figure 13:**
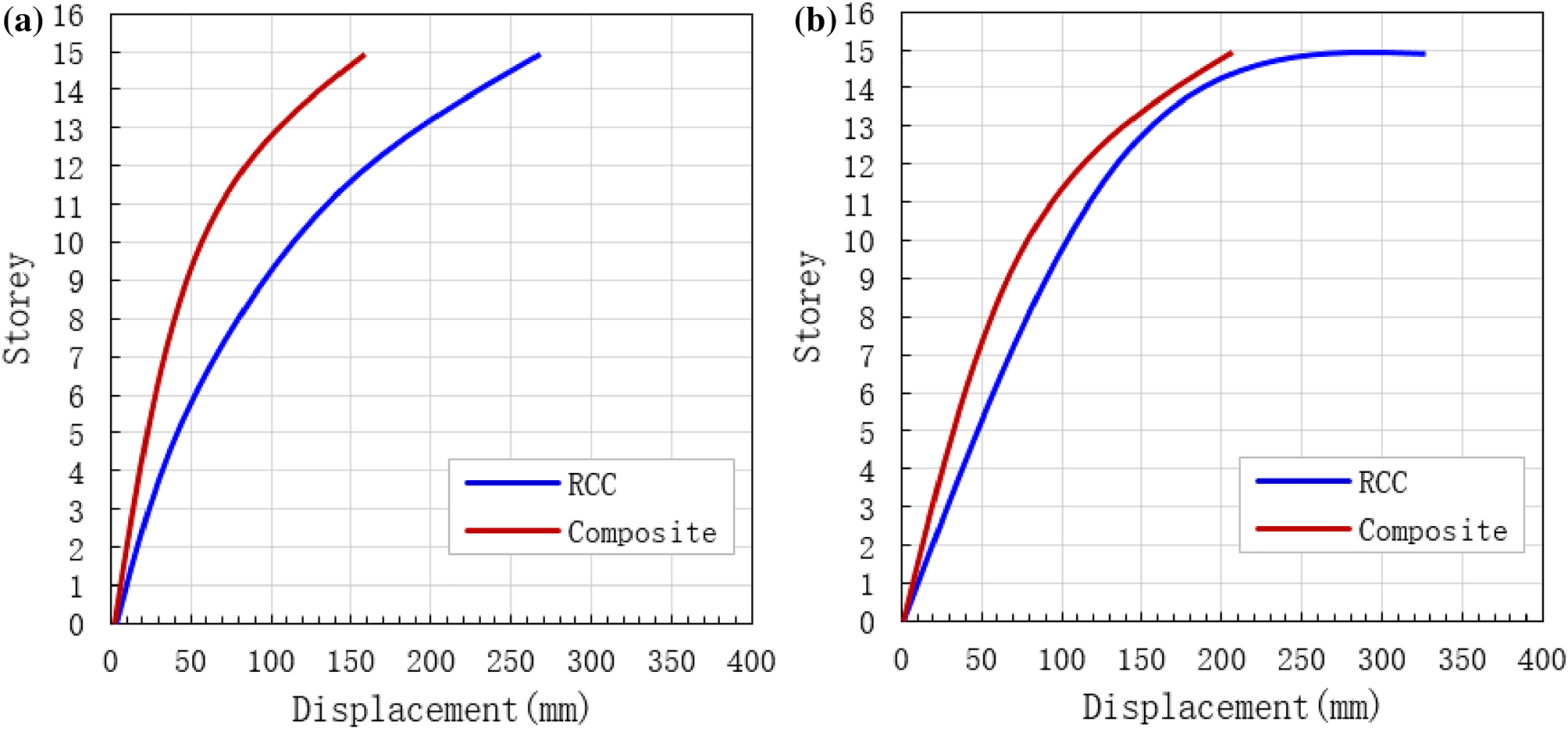
Comparison of storey displacement. (**a**) Under common earthquakes, and (**b**) under rare earthquakes.

Under dynamic loads, the lateral displacement of the RCC and composite structures increased as the number of storey increased (Fig. 13). In the case of common dynamic load, the maximum lateral displacement of RCC and composite buildings were 270 and 160 mm, respectively (Fig. 13a). As the number of floor increases, the lateral displacement also increases rapidly when the structure was subjected to common earthquakes as shown in Fig. 13a. Figure 13b shows the relationship between floor numbers and lateral displacement under rare earthquakes. The maximum top displacement of 330 and 210 mm was recorded for RCC and composite buildings, respectively. It is clear from Fig. 13 that the number of stories has significant effect on structure displacement. Furthermore, the lateral displacement of the RCC model was higher as compared to the composite model.

### Storey drift

**Figure 14:**
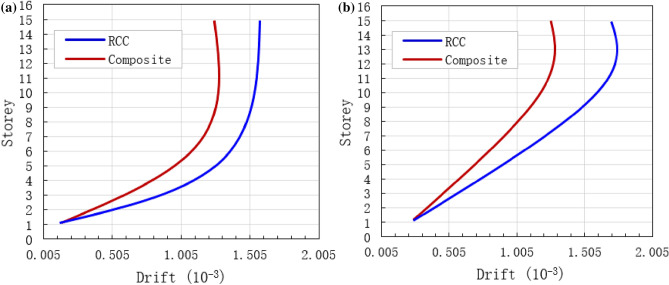
Comparison of storey drifts, (**a**) Under common earthquakes, and (**b**) under rare earthquakes.

Storey drift under seismic loads is also simulated by using a new method within the framework of ABAQUS software. The inter-storey drift of the RCC and composite structures at different vulnerability levels are shown in Fig. 14. The maximum drift in the upper stories of the RCC and composite buildings were 1.25 × 10^–3^ and 1.6 × 10^–3^, respectively, when the models were subjected to common seismic load (Fig. 14a). In case of rare earthquakes, the maximum drift in the upper stories of the RCC and composite buildings were 1.35 × 10^–3^ and 1.78 × 10^–3^, respectively (Fig. 14b). In all cases, the drift of base stories was very small as shown in Fig. 14. This is due to the increase in excitation intensity and soil softness, which leads small value drift in the lower stories. As compared to RCC structure, the storey drift of the composite structure was significantly less at both loading conditions. The inter-storey drift of both RCC and composite structures first increased linearly as the number of storeys increased, and then it rose very rapidly for the top floors.

### Top deflection

**Figure 15:**
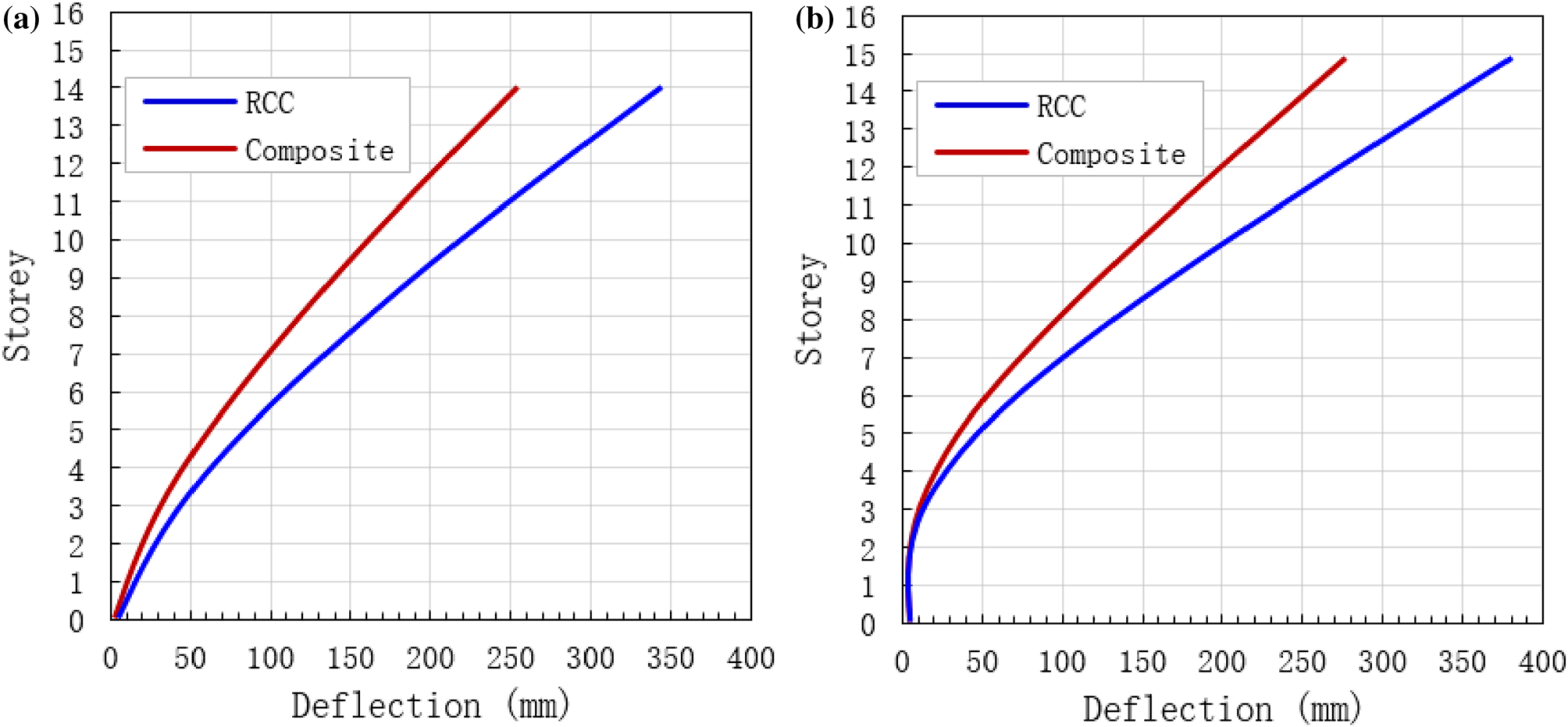
Maximum top structural deflection. (**a**) Under common earthquakes, and (**b**) under rare earthquakes.

A comparison between RCC and composite buildings was conducted to evaluate the benefits of the proposed methodology. Under common and rare earthquakes, the maximum deflection of both RCC and composite buildings is studied through the proposed plastic deformation (PD) method and results are presented in Fig. 15. The deflection of each storey increases linearly as a number of floors increases (Fig. 15). The maximum top deflection of 255 and 340 mm was recorded in RCC and composite buildings under common seismic load, respectively (Fig. 15a). Under rare dynamic loads, the maximum top deflection of 280 and 380 mm was recorded in RCC and composite buildings, respectively (Fig. 15b). Under common and rare seismic loads, the top deflections of the composite building were 33 and 36% higher that of RCC buildings, which shows that the composite mode of construction for tall building subjected to seismic loads is better than RCC building.

According to numerical results, composite members are more durable and resist more loads as compared to to RCC structures under both common and rare dynamic loading conditions. However, many scholars and designers are not welcoming the construction of composite multi-storey buildings due to their complex design analysis, unfamiliarity, and lack of awareness^66,67^. On the other hand, the desirability of the composite mode of construction for tall buildings is due to its effective shear connection between the RCC and structural steel. This is the shear connection that permits both static and dynamic forces to transfer easily into other members^68^. So, shear connections are very important and very basic members for composite construction under static and dynamic loads. Shear connections increase the load resistance ability and overall rigidity of the structural elements (*e.g.,* column and beam).

Furthermore, the proposed method is specifically used for plastic analysis of buildings that show residual stresses. The complementary strain energy method has been confirmed to be effective in a variety of building types, and in the current paper, it is applied as a means of limiting building collapse collapse. Whereas the range of this research is limited by these factors, future work could explore the application of the PD method to other mechanical experimental tests or RCC buildings.

## Conclusions

In this research, a plastic deformation (PD) method is used for a comparative analysis of 15-storey RCC and composite buildings using ABAQUS software. Several interesting parameters, such as; peak acceleration, storey stiffness, lateral displacement, plastic strain, drift, shear force, and deflection were studied for both RCC and composite structures under static and dynamic loads. It was found from the comparative analysis that the use of composite structures in place of RCC structures in construction industry is more durable. Composite structural elements (columns and beams) were found to be the best mode of construction for multi-storey buildings while comparing with the conventional RCC members as they resist more dynamic loads and also serve well for different parameters such as; peak acceleration, stiffness, lateral displacement, storey drift, shear force and deflection. The fundamental time period of the RCC model was found to be 5.2 s while the fundamental time period of the composite model was 6 s. Under common and rare earthquake leads, the peak acceleration of the RCC building was 19% and 22% higher than composite buildings, respectively. The maximum drift of upper stories of the RCC and composite buildings were 1.25 × 10^–3^ and 1.6 × 10^–3^, respectively, when the buildings were subjected to common seismic loads. In the case of rare earthquakes, the maximum drift in the upper stories of the RCC and composite buildings were 1.35 × 10^–3^ and 1.78 × 10^–3^, respectively. The top deflections of the composite building were 33 and 36% higher than those of RCC buildings under common and rare seismic loads, respectively. It was also pointed out that the new plastic deformation method and the large-scale finite element software ABAQUS are suitable for dynamic elastoplastic analysis of tall buildings subjected to seismic loads. Under common and rare earthquakes, the PD method can be effectively used for the analysis of super high-rise structures to achieve the fortification goal without collapsing.

However, there are still many unresolved problems in structural damage assessment through this method, such as the definition of material damage, the combination of displacement and cumulative energy consumption, and the input of seismic waves, which are currently controversial issues and must be investigated in future for deeper understanding.

## Data Availability

The data used to support of this study are included within the article.
